# An Exploration of Ethical Issues in Research in Children’s Health and the Environment

**DOI:** 10.1289/ehp.9000

**Published:** 2006-08-14

**Authors:** Jerome A. Paulson

**Affiliations:** Mid-Atlantic Center for Children’s Health and the Environment, George Washington University School of Public Health and Health Services, Washington, DC, USA

**Keywords:** bioethics, children, community-based research, confidentiality, environmental exposure, environmental health, environmental justice, ethics, financial disclosure, institutional review board

## Abstract

The consideration of ethical issues relating to pediatric environmental health is a recent phenomenon. Discussions of biomedical ethics, research on children, and environmental health research have a longer history. In the late 1990s, researchers at the Kennedy Krieger Institute in Baltimore, Maryland, undertook a study to compare the effectiveness of several methods of reducing lead risk in housing. In a preliminary finding in the case of *Grimes v. Kennedy Krieger Institute, Inc*., a Maryland court questioned the ethics of performing research on children when there is no prospect of direct benefit to those children and whether parents can consent to such research. This case dramatically raised the profile of ethical issues among the pediatric environmental health research community. To broaden the discussion of these issues and in response to the Kennedy-Krieger case, the Children’s Environmental Health Network held a working meeting on 5 and 6 March 2004 to explore this topic. The articles in this mini-monograph were prepared by the authors as a result of the workshop and represent their opinions. This article is an introduction to the workshop and a summary of the articles to follow.

Any discussion of medical ethics in the Western world rests on a foundation that is > 2,500 years old (Jonsen 2000; Pellegrino 1993). The consideration of ethical issues related to research, and specifically research related to environmental health, is a much more recent phenomenon (Soskolne 1993; Soskolne and Light 1996; World Health Organization 1996).

A research project was undertaken by the staff of the Kennedy Krieger Institute Inc. (KKI) in Baltimore, Maryland, to compare different approaches to reducing the risk of lead poisoning in dwelling units. Lawsuits were filed against the researchers and KKI alleging that the children sustained lead poisoning or were put at risk of lead poisoning because the research protocol required that some of the dwelling not be fully abated. In the case of *Grimes v. Kennedy Krieger Institute, Inc*., a court in Maryland rendered a preliminary opinion that nontherapeutic research on children was inappropriate (Court of Appeals of Maryland 2000; Glantz 2002). That opinion caused a great deal of distress and discussion in the environmental heath community (Bellinger and Dietrich 2002; Glantz 2002; Markowitz and Rosen 2002; Mastroianni and Kahn 2002; Mielke 2002; Mushak 2002; Needleman 2002; Nelson 2002; Phoenix 2002; Pinder 2002; Ryan and Farr 2002; Sharpe 2002). Partly in response to the Kennedy-Krieger episode and wanting to expand the discussion about ethical issues related to research in children’s health and the environment, the Children’s Environmental Health Network (CEHN), with funding from the Centers for Disease Control and Prevention and cooperation from the American Association for the Advancement of Science, held a working meeting to explore this topic. Meeting participants were drawn from four constituencies: parents, activists, scientists, and ethicists. (See Appendix 1 for a list of participants and their affiliations.) The articles that follow in this monograph were prepared by the authors as a result of the workshop and represent their opinions.

## Background

The foundations of medical ethics in Western culture go back to writings attributed to Hippocrates and deal primarily with the relationship between individual physicians and their individual patients. There is not much recorded modification of these concepts until the 18th century (Jonsen 2000; Pellegrino 1993; Seto 2001).

The origin of concern about ethical issues related to medical research is not very clear. In the late 19th century, William Osler and others believed that “every treatment is an experiment” (Jonsen 2000). There were discussions of research ethics in the late 19th and early 20th centuries (Vollman and Winau 1996), and distinctions were drawn between research on patients and nontherapeutic research (Katz 1996). At the beginning of the 20th century, medical research as research began to come into its own with the work of Reed, Carroll, Lazear, and Agramonte on the etiology of yellow fever (Jonsen 2000). However, the recognition of the need for a code of ethics governing research emerged after World War II with the discoveries of the so-called medical experiments carried out by Nazi physicians working in death camps. These discoveries led to the Nuremberg Doctor’s Trial in 1947 and the subsequent promulgation of the Nuremberg code (Barondess 1996; Grodin and Annas 1996; Katz 1996; Wiesel 2005). Although the Nazis carried out the worst medical experiments, ethical impropriety occurred in the United States during and after World War II. During World War II, American physicians conducted malaria research on German prisoners housed in prisons in Illinois (Harkness 1996). The Tuskegee syphilis study and the Willowbrook hepatitis study are two glaring examples of research impropriety carried out in the United States after World War II (Seto 2001). In fact, Faden et al. (1996), citing radiation experiments done in the United States in the 1950s, argue that the Nuremberg Code had little impact on medical experimentation until about the 1960s. It was in the 1960s and 1970s that the field of bioethics began to emerge and actively study, report on, and provide guidance regarding ethical issues in research (Callahan and Jennings 2002).

In 1964, the World Medical Association promulgated the Declaration of Helsinki: Ethical Principles for Medical Research Involving Human Subjects. It has subsequently been amended. Among other changes in the subsequent documents has been a deletion of the distinction between therapeutic and nontherapeutic research (World Medical Association 2000).

By the 1970s, there was sufficient pressure in the United States for Congress to pass the National Research Act (1974), which established the National Commission for the Protection of Human Subjects of Biomedical and Behavioral Research. In 1978, the commission published the Belmont Report, titled *Ethical Principles and Guidelines for the Protection of Human Subjects of Research* (National Commission for the Protection of Human Subjects of Biomedical and Behavioral Research 1978). This led to the formulation of Title 45, Code of Federal Regulations, Part 46, “Additional Protections for Children Involved as Subjects in Research,”also known as the Common Rule (Office of Human Subjects Research 2005). This regulation applies to 17 federal agencies.

Subsequently, there was a growing recognition that “special populations,” such as children, minorities, and women, needed either special protection in the research setting or special access to participate in research (National Institutes of Health 1998; Seto 2001). The federal regulations protecting human subjects, 45 CFR 46, has been modified to include special protection and special access for children, and the U.S. Department of Health and Human Services has provided a guidance document on the use of the regulations (Office of Human Subjects Research 1983, 2005). (Subsection D does not apply to the Food and Drug Administration.) A recent Institute of Medicine review of federal protection of children involved in research concluded “that the federal regulations providing special protections for child participants in research are generally appropriate for children of different ages” (Field and Behrman 2004). Various pediatric medical societies have provided recommendations about research involving children [American Academy of Pediatrics 2001, 2004; Etzel and APA (Ambulatory Pediatric Association) Research Committee 2005].

Where public health deals with groups and communities as opposed to individuals, ethical issues in public health are not necessarily the same as the ethical issues in medicine. Likewise, there are comparable distinctions between ethical issues related to public health research compared with biomedical research (Callahan and Jennings 2002; Levin and Fleischman 2002; Thomas et al. 2002). When doing public health research, the focus is generally on groups of individuals and on communities rather than on single individuals. Although the rights of the individuals in those groups and communities need to be scrupulously protected, consideration needs to be given to the protection of the rights of the groups and communities as a whole (Lane et al. 2000; Quinn 2004). Discussions within the field of public health have addressed how to incorporate ethical precepts into practice and research and recognition of the need for increased dialogue such as represented by this CEHN project (Callahan and Jennings 2002).

Because public health research involves communities, it has been argued that communities must be involved in public health research. Community advisory boards have been suggested as one means of achieving this end (Quinn 2004). In many instances, the activities undertaken by public health researchers and those undertaken by public health practitioners are identical: collecting data about communities. One way to distinguish between the two—and thereby determine whether the additional ethical constraints applied to research should apply—is whether the information is to be used for generalization or to be applied to a specific issue (MacQueen and Beuhler 2004). However, participants in a National Center for HIV, STD, and TB Prevention workshop have recommended “the development of alternative methods of oversight for public health investigations that are less dependent on the research versus practice distinction and more geared to assessing the level of risk and ensuring ethical conduct” (MacQueen and Beuhler 2004).

After the CEHN workshop, the National Research Council and the Institute of Medicine convened the Committee on Ethical Issues in Housing-Related Health Hazard Research Involving Children, Youth and Families. The effort was undertaken at the request of the Department of Housing and Urban Development, the Centers for Disease Control and Prevention, and the U.S. Environmental Protection Agency (EPA). Their report, *Ethical Considerations for Research on Housing-Related Health Hazards Involving Children*, was published in 2005 (National Research Council and Institute of Medicine 2005).

In 2004, another controversy erupted that reemphasizes the need for more discussion and debate about ethical issues related to children’s environmental health research. The U.S. EPA proposed the Longitudinal Study of Young Children’s Exposures in their Homes to Selected Pesticides, Phthalates, Brominated Flame Retardants, and Perfluorinated Chemicals. This was also known as the Children’s Health Environmental Exposure Research Study (CHEERS) (U.S. EPA 2005). CHEERS proposed tracking the use of pesticides by families with young children. For a number of reasons—the study seemed to target poor families, some perceived the study as encouraging parents to use pesticides contrary to U.S. EPA guidance, and the study was partly funded by the American Chemistry Council, which represents pesticide manufacturers and other chemical companies—the study came under severe criticism in the professional and lay press (Brumfiel 2004; Eilperin 2004a, 2004b, 2004c; Janofsky 2005; Stokstad and Kaiser 2004). The study was ultimately canceled by the U.S. EPA.

## Workshop Summary

It is within this context and background that the CEHN held its Workshop on Ethical Issues in Children’s Environmental Health Research on 5 and 6 March 2004.

Papers were presented by Maura A. Ryan, Bruce P. Lanphear, Richard R. Sharp, Peggy Shepard, and Celia B. Fisher. The articles in this mini-monograph include the paper prepared by Lanphear, Paulson, and Beirne, with input from meeting attendees; the paper prepared by Ryan; two articles based on Fisher’s presentation; and an article prepared after the meeting by Steven G. Gilbert. The following are highlights from each of these articles.

### Trials and tribulations of protecting children from environmental hazards

Lanphear et al. (2006) argue that regulations to protect children from exposure to environmental hazards are necessary. Although those regulations have improved over the last several decades, they need to be strengthened. In particular, new chemicals must be subjected to premarket testing that includes searching for evidence of reproductive and developmental neurotoxicity. The authors point out that the current system of post hoc identification of problems places the cost of disease, research, and prevention on society while leaving the profits in private hands. Epidemiologic experiments (or randomized controlled trials) are necessary to study various options that may be available for the amelioration of an identified environmental health hazard. The authors suggest seven criteria for conducting environmental health intervention studies (Appendix 2).

A second issue is that a child needs to participate in the decision about whether he or she will participate in a research trial. The National Commission for Protection of Human Subjects of Biomedical and Behavioral Research has set 7 years as a reasonable minimum age for involving children in the assent process, and there are empirical data regarding the ages at which children can understand components of the assent/consent process (see also Fisher 2006a, 2006b).

There is considerable debate about when and how research results should be disclosed to participants. Some criteria have been suggested by the National Bioethics Advisory Commission (1999). One of the foci of the debate is what to do with information for which there are no clinically relevant interpretations. Lanphear et al. (2006) recommend seeking the advice of community representatives and study participants about the resolution of this issue for specific studies (see also Gilbert 2006).

Given that society has developed guidelines for the prior evaluation of a certain class of chemicals before those are placed in the bodies of children (i.e., drugs), there should be comparable guidelines for the evaluations of chemicals that are known to potentially enter children’s bodies (i.e., chemicals used in commerce).

### The politics of risk: ethical issues in children’s environmental health

Ryan (2006) takes as a given children’s increased physical vulnerability to environmental health hazards and points out their differential vulnerability socially, economically, and politically. She calls for the integration of environmental justice standards into the design, implementation, and evaluation of research paradigms. Her essay uses four framing issues to focus the discussion: *a*) the role of social, economic, and racial vulnerability; *b*) vulnerability through invisibility; *c*) the undervaluing of childhood; and *d*) the limits of traditional bioethics paradigm in public health research.

Ryan (2006) points out that children are at risk because of parental choices—and that they are also at risk because of parental lack of choices dictated by race, poverty, and geography. Being poor and having minority status limit choices regarding housing and many other factors influencing exposure to environmental health hazards.

Children’s issues are often ignored or undervalued in the United States. Children do not have a political voice independent from that of their parents, and perhaps as a result, the United States spends far more on services for the elderly than on services for families with children. Research focused on the special needs and vulnerabilities of children is limited. Although children do need special protection in research settings, it is important not to use children’s special status to prevent research directed to children’s needs, such as the prevention of environmental health problems.

Ryan (2006) raises the thorny questions about the role of market or utility-oriented cost–benefit pressures in and on children’s environmental health research and the issue of discounting—that is, using cost–benefit analysis to assess environmental impact by applying a discount rate to future costs and benefits, in the formulation of environmental health policy. How does one “weigh risks associated with participation in environmental health research against risks already existing in the potential subject’s everyday home environment and [identify] what monitoring tools best signal timely evidence of unacceptable risk[?]” (Ryan 2006). By using economic discounting over the lifespan of a child, one can rapidly reach the conclusion that environmental interventions are never cost-effective.

Finally, Ryan (2006) points out that many of the paradigms used in traditional biomedical research do not fit when one is trying to do community-based public health research. Ethical considerations need to adapt to protect children and communities while granting them input to the research design and access to the research results.

### Privacy and ethics in pediatric environmental health research: genetic and prenatal testing, and protecting families and communities

Fisher (2006a, 2006b) is concerned that current federal regulations and organizational standards may not be responsive to the ethical issues raised by recently introduced methodologic approaches to children’s environmental health research, such as community-based research (CBR). She believes that environmental health research needs to create an ethical framework that is robust enough to protect the privacy rights of children and families participating in protocols irrespective of methodology used, the age of the children involved, or the type of population being studied.

The use of information is a two-edged sword: possibly leading to the remediation of environmental health problems or possibly leading to the stigmatization of individuals or communities. Ethical challenges are compounded when poor and less powerful populations are recruited for environmental hazards research. Attention to genetic susceptibility and cultural practices associated with environmental disease in underserved groups can unintentionally promote existing health disparities by placing responsibility on the population rather than environmental policies.

Environmental health research may collect or analyze data over a very long term; for example, the National Children’s Study (2006) is projected to collect data for at least 21 years, and analysis will continue for decades beyond that (Branum et al. 2003). This makes it impossible to predict all of the analyses that will be done and to get appropriate consent in advance. The changing status of the child over the length of the study gives the child different rights and protections at different times. At certain points in their lives, children have neither the legal status nor cognitive or experiential status to understand the privacy implications of research involvement. At other times, they do have this status.

Where genetic data are collected, information may be able to be inferred about family members who are not actually subjects in the study. This puts the privacy of those individuals at risk. It is also unclear whether and how genetic information should be shared with parents and subsequently with children as they grow. What does one do with information about the predisposition to a problem that may develop as the result of exposure to an environmental contaminant? Some have recommended that parents not have access to information derived from the analysis of their child’s DNA unless it reveals a genetic condition that can be ameliorated, prevented, or treated before the child reaches the age of majority. Others recommend that genetic information obtained through research should be disclosed only if the hereditary nature of environmental disease susceptibility has been clearly demonstrated, the disease presents a major risk to the child’s future health, there is a low probability of false positives, and remedies are possible.

Under current federal regulations—the Common Rule (Office of Human Subjects Research 2005)—the collection of identifiable private information defines someone as a human subject. Fisher (2006a, 2006b) discusses the debate among ethicists as to what constitutes private information. She points out that divulging such information has potential adverse personal, employment, and economic impacts on individuals.

Fisher (2006a, 2006b) points out that, in general, parents or guardians can give consent for children to participate in research, and federal regulations call for obtaining assent from older children. Studies have indicated that children have the necessary cognitive ability to provide informed consent by mid-adolescence. However, perception of pressure for parents and/or researchers may prevent adolescents from exercising their rights. Fisher recommends that researchers proactively educate child-subjects throughout the course of the research project in age-appropriate fashions about the research, its risks, its benefits, information disclosure, and other issues.

There are cultural differences in attitudes about sharing information within families and with the general public. Researchers need to be cognizant of these issues and incorporate that information into the process of describing issues to families and of obtaining assent and consent.

Adults have a “right not to know” about their susceptibility to disease when early treatment is not available. However, it is not clear whether parents have a similar right regarding potential disease in their children.

When data are collected perinatally, such as through cord blood samples, the information revealed can include facts and assessments that the mother may have wished to keep private. Revelation of this information may, among other outcomes, lead to criminal investigation, a child welfare complaint, loss of food stamps, or loss of Supplemental Security Income. Data on maternal exposure to or ingestion of teratogenic agents also raise questions regarding the rights of other family members to this information. Does the child’s biologic or legal father have the right to information about maternal environmental exposure? Subsequently, do adult children with congenital anomalies or other health problems associated with maternal exposures or ingestion of environmental toxicants have the right to know about their mother’s environmental history if the information is available in data banks? The burden of determining whether to give permission for the use of this information falls disproportionately on women.

Researchers, institutional review boards (IRBs), and other responsible parties need to be mindful of the question “Who is a human subject?” Federal regulations define a “human subject” as a living individual about whom an investigator obtains data through intervention or interaction with the individual or about whom the investigator has recorded individually identifiable private information. What happens when the primary subject (e.g., the child or adolescent) is asked for information about the home environment that may elicit personal data about family members? What happens when genetic studies may reveal facts about family members other that the identified subject? Would those family members be stigmatized?

Whether a family member is protected under federal regulations for human subjects research thus depends upon whether the data include unique individual identifiers (e.g., name, address, social security number) or information from which the family member’s identity can be easily ascertained.

Fisher (2006a, 2006b) deals with the issue of withdrawal from studies and the particular impact that this may have on longitudinal environmental health research. Data from cross-sectional studies are easier to anonymize. During longitudinal studies, the link between the data and the subject must be maintained until all analyses are complete. She asserts that there is little scientific or public consensus on whether individuals ought to be permitted to withdraw previously collected research samples if they exert their right to withdraw from the study. She maintains that the Health Insurance Portability and Accountability Act (HIPAA 1996), if the protected health information has already been obtained and used on the basis of the original authorization, allows the investigator to maintain data analysis based on the information, although no additional information may be used or disclosed after revocation. She also notes that it may be ethically appropriate to permit the withdrawal of data held in data banks at the participant’s request, but that this policy need not extend indefinitely.

Fisher points out that maintaining privacy can be very difficult, if not impossible, when the community involved is very small and or the toxicant involved is very unusual.

She also raises the question of whether individuals from minority communities and the minority communities themselves are disproportionately burdened with the issues discussed because they are, in fact, disproportionately burdened with exposure to environmental toxicants (see Ryan 2006).

### Supplementing the traditional IRB with an environmental health and community review board

Gilbert (2006) proposes the development of an environmental health and community review board (EHCRB) that would function as a traditional IRB but with added expertise and focus related to concerns of the community. He posits that this is necessary because CBR often involves additional ethical, legal, and social considerations beyond those of the specific individuals involved in the study. The current IRB system was established to protect living, individual research subjects. CBR involves many community members in the planning and execution of the research, and by definition, the research must benefit the community. Performing CBR involves the community and emphasizes social responsibility and partnership. Therefore, the traditional IRB structure, with its emphasis on the individual, needs modification.

The dignity and worth of the individual must be respected and recognized. This implies that beyond the right to know, there is a right to understand. This respect transcends the individual and extends to the family and the community. Researchers have a duty to provide the community with all of the facts in their possession and to work with the community to determine what is good or not harm. Researchers must ensure that the work that they undertake with the community is sustainable; that is, it must not harm the community, and it must improve the community’s functionality and capacity.

The notion of justice must also be expanded to include the community. There will inevitably be conflicts among the notions of justice at the individual, corporate, and community levels, but the needs and interests of the community must have primacy. The concept of environmental justice as defined by the U.S. EPA (2006) and used widely in the realm of environmental health research supports the creation of an EHCRB. The EHCRB would evaluate the implications of the study for the families and the community involved, as well as the individuals. In doing research, researchers may need to obtain consent from the family and/or community unit in addition to the individuals involved. The EHCRB must determine who is in the community and take into consideration the needs of all the stakeholders in the community. It must balance the different interests and concerns of the various components of the community—citizens, businesses, corporations, and governments—over time.

An EHCRB must include individuals knowledgeable about and representative of the community where the research will be done. The EHCRB should be based in the community.

## Summary

It is a given that children are not little adults when it comes to their anatomy, physiology, and psychology. It is also clear from this workshop that the regulations that have been devised to protect individual adult research subjects do not meet all of the needs of children and do not meet the needs of communities that may also be “research subjects.” The workshop has developed a set of proposed criteria for conducting environmental health intervention trials involving children. More attention needs to be paid to privacy issues in environmental health research. The concern for the privacy of the individual remains, but attention needs to be paid to the privacy of family members when genetic or other related information is part of the project, and attention needs to be paid to the privacy of other community members. Involvement of the community, through the creation of EHCRBs, will provide an added layer of protection to this broader concept of the research subject and will also involve the community as active participants in the research. The proposed National Children’s Study (2006) includes a working group on ethics. It is our hope that the fruits of this project will inform the National Children’s Study efforts and the efforts of others involved in children’s environmental health research.

## Appendix 1. Conference Participants

**Table:**

Name	Organization
Whitlynn Battle	Citizens’ Lead Education and Poisoning Prevention, Birmingham, AL
Victoria Baxter	American Association for the Advancement of Science, Washington, DC
Sandra Beirne	University of Washington, Seattle, WA
Joy Carlson	J. Carlson Consulting, Oakland, CA
Audrey Chapman	American Association for the Advancement of Science, Washington, DC
Marilyn Field	National Academy of Sciences, Washington, DC
Celia Fisher	Fordham University, Bronx, NY
Maida Galvez	Mount Sinai School of Medicine, New York, NY
Marcheta Gilliam	Legal Aid, Cincinnati, OH
Nathan Graber	Mount Sinai School of Medicine, New York, NY
Brenda Gross	Families in Search of Truth, Fallon, NV
Nancy Halpern Ibrahim	Esperanza Community Housing Corporation, Los Angeles, CA
David Jacobs	U.S. Department of Housing and Urban Development, Washington, DC
Bruce Lanphear	Cincinnati Children’s Hospital Medical Center and the University of Cincinnati, Cincinnati, OH
Mary Lawson	Earth Seed, Baltimore, MD
Dee Lewis	Concerned Residents Initiative, Sacramento, CA
Sunday Morning	Earth Seed, Baltimore, MD
Mary Ellen O’Connell	National Academy of Sciences, Washington, DC
Jerome A. Paulson	Children’s Environmental Health Network, Washington, DC
Ashley Peter	U.S. Department of Housing and Urban Development, Washington, DC
John Rosen	Albert Einstein College of Medicine, New York, NY
Jane Ross	National Academy of Sciences, Washington, DC
Charles Rotimi	Howard University, Washington, DC
Don Ryan	Alliance for Healthy Homes, Washington, DC
Maura Ryan	University of Notre Dame, Notre Dame, IN
Jennifer Sass	Natural Resources Defense Council, Washington, DC
Richard Sharp	Baylor College of Medicine, Houston, TX
Rabbi Daniel Swartz	Children’s Environmental Health Network, Washington, DC
Leonardo Trasande	Mount Sinai School of Medicine, New York, NY
Bernard Weiss	University of Rochester, Rochester, NY
Nsedu Obot Witherspoon	Children’s Environmental Health Network, Washington, DC

## Appendix 2. Potential Criteria for the Conduct of Experimental Trials Involving Children

- Examines research questions that cannot be answered by studying adults.
- Addresses uncertainty about the safety or efficacy of environmental interventions.
- Studies an uncertain causal relationship between an exposure and a disease.
- Includes an adequate sample size to test a hypothesis.
- Includes mechanisms for communicating research findings to participants.
- Involves community involvement in the design and implementation of the study.
- Includes mechanisms to ensure participants are fully informed about the rationale for the trial. (Lanphear et al. 2006).
